# Combined treatment of a solid tumour by local hyperthermia and actionomycin D.

**DOI:** 10.1038/bjc.1978.121

**Published:** 1978-05

**Authors:** A. Yerushalmi

## Abstract

(BALB/c X C57BL/6)F1 mice bearing an SV-40 fibrosarcoma in the limb were injected with 0.045, 0.09 and 0.18 microgram/g body wt actinomycin D into the tumour. Similar animals were also treated with local hyperthermia (39.7 degrees, 42.3 degrees and 43.6 degrees C intratumour temperature for 30 min). The marked increase in median survival time following the combination of drug and local hyperthermia indicates that the combined treatment has a synergistic effect in the control of solid tumours. The median survival time of animals receiving the two treatments in immediate succession was higher than in animals with 30 min between treatments. This has important implications for the therapy of human cancer.


*Br. J. Cancer* (1978) **37**, 827

### COMBINED TREATMENT OF A SOLID TUMOUR BY LOCAL HYPERTHERMIA AND ACTINOMYCIN D

A. YERUSHALMI

*From the Radiation Unit, The Weizmann Institute of Science, Rehovot, Israel*

Received 24 May 1977 Accepted 13 January 1978

**Summary.—(BALB/c $\times$ C57BL/6)$F_1$ mice bearing an SV-40 fibrosarcoma in the limb were injected with 0·045, 0·09 and 0·18 $\mu$g/g body wt actinomycin D into the tumour. Similar animals were also treated with local hyperthermia (39·7°, 42·3° and 43·6°C intratumour temperature for 30 min). The marked increase in median survival time following the combination of drug and local hyperthermia indicates that the combined treatment has a synergistic effect in the control of solid tumours. The median survival time of animals receiving the two treatments in immediate succession was higher than in animals with 30 min between treatments. This has important implications for the therapy of human cancer.**

THE inability to produce satisfactory results using single therapeutic agents in patients with advanced cancer led to their use in combinations (De Vita and Schein, 1973). Complete remissions are rare with single agents used against solid tumours, and even when complete remissions are achieved with single agents, their duration is generally short (De Vita, Young and Canellos, 1975).

Excessive toxicity of actinomycins, including actinomycin D (ActD), is the limiting factor in the use of these antibiotics in cancer chemotherapy. ActD is active in the reduction of trophoblastic tumours (Hertz, Ross and Lipsett, 1964), germinal cell neoplasms (Li *et al.*, 1960), soft-tissue sarcomas (Einhorn, 1976) and Wilms' tumour, and as an adjuvant to radiotherapy (Wolf *et al.*, 1968). However, ActD is cytotoxic at an *in vivo* concentration of $LD_{10}$ (0·6 $\mu$g/g i.p.) in mice (Morasca *et al.*, 1974). It was found that, *in vitro*, non-toxic concentrations of ActD enhanced radiation damage by 10–20% (Elkind *et al.*, 1967). A critical limitation of chemotherapy is the fact that a high toxic drug concentration must reach poorly vascularized areas of the tumour in order to produce a beneficial therapeutic effect. In order to circumvent the cytotoxic effects of single-drug therapy, combinations of several agents have been tried. Most of the combinations were based on the differential toxicities of the drugs given separately.

Hyperthermia is more deleterious to malignant than to normal tissue (Kim, Kim and Hann, 1974; Muckle and Dickson, 1971; Dickson and Suzangar, 1976). Hyperthermal enhancement of the sensitivity of cancer cells to ionizing radiation has been demonstrated in humans (Selawry, Carlson and Moor, 1958; Cocket *et al.*, 1967; Shoulders *et al.*, 1942; Warren, 1935; Brenner and Yerushalmi, 1975), in experimental implanted tumours (Robinson, Wizenberg and McCready, 1974; Crile, 1963; Hahn, Alfieri and Kim, 1974; Yerushalmi, 1975, 1976*a*) and in cells in culture (Chen and Heidelberger, 1969; Ben-Hur, Elkind and Bronk, 1974). Hyperthermia has also been shown to act as a sensitizer with cytotoxic drugs (Dickson and Suzangar, 1974). Most reports dealing with the combination of elevated temperature and chemotherapeutic drugs were connected with the investigation of tumour cell-lines *in vitro* (*i.e.*, disconnected from the host). In order to achieve conditions as close as possible to the *in vivo* situation, I selected a solid tumour, the SV-40 fibrosarcoma, and treated it locally in the host. I present results relative to the sensitizing effects of hyperthermia and single, low, non-toxic concentrations of ActD. Hyperthermia potentiated ActD in (BALB/c$\times$C57BL/6)$F_1$ mice bearing the SV-40-induced fibrosarcoma, and the synergistic effect of hyperthermia and ActD was demonstrated.

The criterion of survival prolongation was chosen to assess results, since it is clinically the most significant, taking into account all the treatment effects. This is, of course, the actual goal of every experimental and clinical trial in human patients.

### MATERIALS AND METHODS

(BALB/c$\times$C57BL/6)$F_1$ hybrid mice were used throughout the experiments. The tumour was an SV-40 fibrosarcoma maintained by repeated passage. The tumour is positively detected 5–7 days after transplantation, and causes death within 30–40 days in untreated animals. Spontaneous cures and metastases were not seen during the experimental period in untreated animals. The details of the preparation of tumour and cell suspensions have been described elsewhere (Yerushalmi, 1975). $10^6$ viable tumour cells in 0·5 ml were injected i.m. into the left hind leg. Ten to 11 days after transplantation, animals with tumours of the same volume (1000–1100 $mm^3$) were mixed together, randomly selected, and separated into the various treatment groups. All animals were anaesthetized with an i.p. injection of Pentobarbital Sodium. Blood supply to the limb was not impaired during treatment.

*Heating system.*—The heating system has been described in detail elsewhere (Yerushalmi, 1976*a*). In short, it consisted of a hot-air blower, a heat-insulated cylinder and a temperature-control unit. For local heating, the tumour bearing limbs of 4 mice at a time were inserted into 4 holes drilled in the upper part of the cylinder.

*Temperature measurements.*—The hot-air atmosphere was achieved by passing hot air through an attenuator to reduce turbulence. Homogeneity was achieved, and confirmed by a system of valves and thermocouples installed for temperature stability and control. Simultaneous temperature measurements were made in the cylinder, in the tumour (to record intratumour temperature) and in the ear of the mouse (to record skin temperature). Preliminary experiments showed that intra-ear temperature measurements were reliable indicators of skin temperature (Benzinger and Taylor, 1963; Yerushalmi, 1976*b*). Measurements were taken by copper constantan thermocouples (1·0 mm thick) connected to an electronic control unit which included a continuous digital temperature read-out and an X–Y recorder (Yerushalmi, 1976*a*, *b*).

Intratumour and body-temperature profiles are given elsewhere (Yerushalmi, 1976*a*). Local heating of the tumour-bearing leg with 45°C hot air for 30 min produced an intratumour temperature of 39·7$\pm$0·2°C. Local heating at 50°C for 30 min produced a 42·3$\pm$0·2°C intratumour temperature. Local heating at 55°C hot air for 30 min raised intratumour temperature to 43·6$\pm$0·2°C. Local heat treatment at the highest heating temperature, namely 55°C hot air raised the animal's body temperature to 37·8°C, but this was well tolerated by the treated animals. Heating time includes heat build-up in the tumour ($\sim$12 min).

ActD used was AMD-Cosmogen from Merck, Sharp and Dohme. The drug was dissolved in sterile water and diluted to the treatment concentrations. In all the experiments, the drug was injected into the tumour. The 3 concentrations used were 0·045, 0·09 and 0·18 $\mu$g/g body wt. The lowest value of 0·045 $\mu$g/g was chosen because combined *in vivo* radiotherapy and chemotherapy schedules of 0·05 $\mu$g/g had been used previously (Tomashefsky *et al.*, 1976). ActD at 0·5 $\mu$g/g inhibits the formation of ribosomal subunits in rat liver (Woodscock and Mansbridge, 1971) and the $LD_{50}$ after i.p. injection was found to be 0·59$\pm$0·17 $\mu$g/g (Rahman *et al.*, 1974).

The median survival time (MST) was calculated by the equation $MST = L + (cj/fm)$, where L is the lower day boundary of the group containing the median animal, $j$ is the number of deaths needed to reach the median animal in the median group, $fm$ is the number of deaths in the median group, and $c$ is the group interval (= 1 in these experiments) (Miller, 1973).

### RESULTS

Drug cytotoxiocity was tested at the 3 concentrations used. Healthy control animals were injected i.p. and into the left hind leg. No cytotoxic effects were observed at these concentrations. In normal and tumour-bearing, locally heated animals, no leg losses and no deaths were caused by the heat treatment. There was no skin injury, except for slight hair discoloration. The MST of every group treated by the combined drug plus heat treatment is compared to its untreated control and drug only groups.

The effects of varying drug doses, degrees of tumour hyperthermia, and the combinations at varying intervals on survival of tumour-bearing animals are presented in Tables I and II.

The values in Table III show that, in general, for each of the 3 drug doses tested, there is a temperature-dependent increase in the EMSTR (Enhancement of MST ratio).

TABLE I.—*Median Survival Time (MST) of Tumour-bearing Animals Treated with ActD and ActD+Local Hyperthermia (in Parentheses, No. of Animals in Group)*

| | ActD only | ActD+ local heat (a) | ActD twice (b) |
|---|---|---|---|
| Untreated controls | 40 (10) | — | 39 (13) |
| 0·045 μg/g | 44 (12) | — | 42 (14) |
| 0·09 μg/g | 43 (13) | — | 46 (16) |
| 0·18 μg/g | 41 (10) | — | 44 (12) |
| | | 39·7°C | |
| Untreated controls | 39 (9) | | |
| 0·045 μg/g | 41 (10) | 41 (10) | |
| 0·09 μg/g | 42 (10) | 44 (10) | |
| 0·18 μg/g | 47 (11) | 43 (10) | |
| | | 42·3°C | |
| Untreated controls | 39 (9) | | |
| 0·045 μg/g | 43 (10) | 47 (11) | |
| 0·09 μg/g | 44 (10) | 44 (12) | |
| 0·18 μg/g | 43 (10) | 48 (10) | |
| | | 43·6°C | |
| Untreated controls | 40 (10) | | |
| 0·045 μg/g | 42 (9) | 61 (12) | |
| 0·09 μg/g | 44 (10) | 89 (10)* | |
| 0·18 μg/g | 46 (10) | 54 (11) | |

(a) Schedule: injection of ActD, followed at once by local hyperthermia at the indicated tumour temperature.
(b) 2 injections at an interval of 2 days.
(*) 2 animals cured 275 days after treatment.

TABLE II.—*Median Survival Time (MST) of Tumour-bearing Animals Treated with ActD and with ActD+Local Hyperthermia (in Parentheses, No. of Animals in Group)*

| Dose of ActD | Schedule (a) | Intra-tumour temp. 42·3°C | Intra-tumour temp. 43·6°C | No heat |
|---|---|---|---|---|
| — | | | | 41 (18) |
| 0·045 μg/g | | | | 44 (9) |
| — | | 41 (9) | 42·5 (9) | |
| 0·045 μg/g | H, 0, I | 48 (8) | 51 (9) | |
| 0·045 μg/g | H, 30, I | 45 (8) | | |
| 0·045 μg/g | I, 30, H | 41 (8) | 40 (9) | |
| 0·09 μg/g | H, 0, I | 55 (8)* | 65 (7)‡ | |
| 0·09 μg/g | H, 30, I | 49 (8) | | |

(a) H (heat) or I (injection) with interval in minutes.
(*) One animal cured, 195 days after treatment.
(‡) One animal cured, 210 days after treatment.

TABLE III.—*Temperature-dependent Increase in EMSTR When Heat Immediately Follows Drug*

$$EMSTR = \frac{\text{MST of drug+heat-treated animals}}{\text{MST of drug-treated animals}}$$

| Drug dose (μg/g) | Intratumour temperature, °C 39·7 | 42·3 | 43·6 |
|---|---|---|---|
| 0·045 | 1 | 1·09 | 1·45 |
| 0·09 | 1·05 | 1 | 2·02 |
| 0·18 | 0·92 | 1·12 | 1·17 |

### DISCUSSION

The curative limits of single-drug chemotherapy are well known. Moreover, the side effects of most cancer chemotherapy drugs appear to be dose-dependent. ActD toxicity was found to be cumulative over a wide range of dose schedules in mice and rats (Goldin and Johnson, 1974). Therefore, any possible method which allows the reduction of total drug dose and its toxicity, while achieving the same tumour-cell kill, is clinically relevant. The toxic, hyperthermic, and the combined heat+drug effects on tumour-bearing animals cannot be evaluated in an *in vitro* system.

*In vitro* studies have shown that raised temperatures resulted in immediate but reversible increased diffusion of low-mol.-wt compounds through the cell membrane (Storm *et al.*, 1975) and the synergism of antibiotics with hyperthermia was demonstrated (Mondovi *et al.*, 1969*a*, *b*). Moreover, small differences in the conditions of *in vitro* experiments demonstrated results due to factors other than the heating effects (Mondovi *et al.*, 1969*a*).

It is known that cells from the same tumour behave differently *in vitro* and *in vivo*. In the tumour *in vivo* a non-proliferating fraction is a part of the cell population. Therefore, *in vitro* partial cell kill, due to hyperthermia or hyperthermia combined with drugs, may indicate only the response of the fast-proliferating cell fraction of the tumour, which is dependent on heating time. However, cells that are not fully destroyed by heat recover. This important cell fraction exists in the tumour *in vivo*, and the success or failure in destroying these cells can be monitored only *in vivo*, by the observation of tumour growth.

Furthermore, alterations in respiration and glycolysis, inhibition of DNA, RNA, and protein synthesis *in vitro*, due to heat, could be only a partial cause of the destruction of tumour cells. Thus, extrapolation to the *in vivo* reality is irrelevant, because of the host–tumour relationships missing from the *in vitro* situation. It should be emphasized that there is no information comparing the *in vitro* hyperthermic response of human tumours with the response of the same tumours in the patient.

Therefore, the importance of *in vitro–in vivo* studies in heat damage to tumour growth was emphasized (Dickson and Suzangar, 1974).

The local treatment of a solid tumour, by injecting the drug into the tumour, and local heating of the tumour, has the advantage of taking into account all the above-mentioned effects in the host, while treating a defined mass of tumour cells.

The purpose of the present investigation was to examine the synergism of local hyperthermia and ActD in the control of local tumour growth, and thus reduce the concentration of ActD. Drug administered immediately before or after local hyperthermia increased tumour-control, demonstrating the synergism of local hyperthermia and ActD. In general, drug injected 30 min before or after heating resulted in less tumour control than the immediate combinations of injection and heating, in either order. In parallel experiments (unpublished) the drug was allowed to stay at 37°C for $\frac{1}{2}$ h, injected and immediately heated or heated and immediately injected, with the same results as in Tables I–III. Therefore, artefacts due to changes in drug activity can be ruled out, and the increased tumour control in the immediate combinations of treatments may be attributed to the combined effect of heat + drug. Again, it should be borne in mind that all the treatments consisted of a single low dose of ActD combined with local heating. Therefore, the failure to increase the cure rate does not rule out the possible usefulness of this method.

In the present investigation, I showed that a single, low, nontoxic dose of ActD, combined with local tumour heating, provides a more effective control of local tumour growth than either agent used singly, and that local heating enhances the action of ActD in tumour control, probably by allowing a higher concentration of the drug to reach the hypoxic tumour cells. Low concentrations of AMD in poorly vascularized hypoxic sites may therefore be clinically effective when combined with heat. A single treatment of the combined heat + chemotherapy markedly prolonged the MST and even produced some cures, when compared to ActD-treated animals. The most significant results were obtained with the 43·6°C local intratumour temperature combined with the 0·09 μg/g drug treatment, and there was a temperature-dependent increase in EMSTR at each of the 3 drug doses tested.

The marked increase in life span after the combined drug + local hyperthermia therapy indicates that the method may be useful in solid tumour therapy. Furthermore, data on optimal drug dose, heating temperature and duration of local heating, are critical in the design of this treatment regimen, and investigations similar to these, with the combination of simultaneous heat and ionizing radiation (Yerushalmi, 1976*a*) should be performed.

The author wishes to thank Dr G. Yagil for helpful suggestions during the preparation of this manuscript.

REFERENCES

BEN HUR, E., ELKIND, M. M. & BRONK, B. V. (1974) Thermally Enhanced Radio-response of Cultured Chinese Hamster Cells. Inhibition of Repair of Sublethal Damage and Enhancement of Lethal Damage. *Radiat. Res.*, **58**, 38.

BENZINGER, T. H. & TAYLOR, G. W. (1963) Cranial Measurements of Internal Temperature in Man. In *Temperature: Its Measurement and Control in Science and in Industry*, vol. 3. Ed. C. M. Herzfeld, New York: Reinhold Publ. p. 111.

BRENNER, H. J. & YERUSHALMI, A. (1975) Combined Local Hyperthermia and X-irradiation in the Treatment of Metastatic Tumors. *Br. J. Cancer*, **39**, 91.

CHEN, T. T. & HEIDELBERGER, C. (1969) Quantitative Studies on Malignant Transformation of Mouse Prostate Cells by Carcinogenic Hydrocarbons *in vitro*. *Int. J. Cancer*, **4**, 166.

COCKET, A. T. K., KAZIM, M., NAKAMURA, R., FINGERHUT, A. & STEIN, J. J. (1967) Enhancement of Regional Bladder Megavoltage Irradiation in Bladder Cancer, using Local Bladder Hyperthermia. *J. Urol.*, **97**, 1034.

CRILE, G. (1963) The Effect of Heat and Radiation on Cancer Implanted in the Feet of Mice. *Cancer Res.*, **23**, 372.

DE VITA, T. V. & SCHEIN, P. S. (1973) The Use of Drugs in Combination for the Treatment of Cancer. Rationale and Results. *New Engl. J. Med.*, **288**, 998.

DE VITA, T. V., YOUNG, R. C. & CANELLOS, G. P. (1975) Combination *versus* Single Agent Chemotherapy: A Review of the Basis for Selection of Drug Treatment of Cancer. *Cancer*, **35**, 98.

DICKSON, J. A. & SUZANGAR, M. (1974) *In vitro* and *in vivo* Studies on the Susceptibility of the Solid Yoshida Sarcoma to Drugs and Hyperthermia (42 degrees). *Cancer Res.*, **34**, 1263.

DICKSON, J. A. & SUZANGAR, M. (1976) In: *Organ Culture in Biomedical Research* (British Society for Cell Biology Symposium, I). Eds. Balls and Monnickendam. Cambridge Univ. Press. p. 417.

EINHORN, L. H. (1976) General Principles of Cancer Chemotherapy, *Connecticut Med.*, **40**, 159.

ELKIND, M. M., KAMPER, C., MOSES, W. B. & SUTTON-GILBERT, H. (1967) Sublethal Radiation Damage Repair in Mammalian Cell Recovery and Repair Mechanism in Radiobiology. *Brookhaven Symposia in Biol.* **20**, 134.

GOLDIN, A. & JOHNSON, R. K. (1974) Evaluation of Actinomycins in Experimental Systems. *Cancer Chemother. Rep.*, **58**, 63.

HAHN, E., ALFIERI, A. A. & KIM, J. H. (1974) Increased Cures using Fractionated Exposures of X-irradiation and Hyperthermia in the Local Treatment of the Ridgeway Osteogenic Sarcoma in Mice. *Radiology*, **113**, 199.

HERTZ, R., ROSS, G. T. & LIPSETT, M. B. (1964) Chemotherapy in Women with Trophoblastic Disease: Choriocarcinoma, Chorioadenoma Deustruens and Complicated Hydatidiform Mole. *Ann. N.Y. Acad. Sci.*, **114**, 88.

KIM, J. H., KIM, S. H. & HANN, E. W. (1974) Thermal Enhancement of Radiosensitivity, using Cultured Normal and Neoplastic Cells. *Am. J. Roentgenol.*, **121**, 860.

LI, M. C. *et al.* (1960) Effects of Combined Drug Therapy on Metastatic Cancer of Testis. *J. Am. Med. Assoc.*, **174**, 1291.

MILLER, I. (1973) Instruction 14, Automated Information Section, Drug Evaluation Branch, DR & DP, DCT, NCI.

MONDOVI, B., STORM, R., ROTILIO, G., FINAZZI-AGRO, A., CAVALIERE, R. & ROSSI-FANELLI, A. (1969*a*) The Biochemical Mechanism of Selective Heat Sensitivity of Cancer Cells, I. Studies on Cellular Respiration. *Eur. J. Cancer*, **5**, 129.

MONDOVI, B., FINAZZI-AGRO, A., ROTILIO, G., STORM, R., MORICCA, G. & ROSSI-FANELLI, A. (1969*b*) The Biochemical Mechanism of Selective Heat Sensitivity of Cancer Cells. II Studies on Nucleic Acid and Protein Synthesis. *Eur. J. Cancer*, **5**, 137.

MORASCA, L., BALCONI, G., EBRA, E., LELIVELD, P. & VAN PUTTEN, L. M. (1974) Cytotoxic Effects *in vitro* and Tumor Volume Reduction *in vivo* Induced by Chemotherapeutic Agents. *Eur. J. Cancer*, **10**, 667.

MUCKLE, D. S. & DICKSON, J. A. (1971) The Selective Inhibitory Effect of Hyperthermia on the Metabolism and Growth of Malignant Cells. *Br. J. Cancer*, **25**, 771.

RAHMAN, Y. E., CERNY, E. A., TOLLAKSEN, S. L., WRIGHT, B. J., NANCER, S. L. & THOMSON, J. F. (1974) Liposome-encapsulated Actinomycin D: Potential in Cancer Chemotherapy. *Proc. Soc. exp. Biol. Med.*, **146**, 1173.

ROBINSON, J. E., WIZENBERG, M. J. & MCCREADY, W. A. (1974) Radiation and Hyperthermal Response of Normal Tissue *in situ*. *Radiology*, **113**, 195.

SELAWRY, O. S., CARLSON, J. C. & MOOR, G. E. (1958) Tumor Response to Ionizing Rays at Elevated Temperatures. *Am. J. Roentgenol.*, **80**, 833.

SHOULDERS, H. S., TURNER, E. L., SCOTT, L. D. & GRANT, W. H. (1942) Effect of Combined Fever and X-ray Therapy on Far-advanced Malignant Growths. *Radiology*, **39**, 184.

STORM, R., CRIFO, C., BOZZI, A. & ROSSI-FANELLI, A. (1975) Inhibition by Elevated Temperatures of Ribosomal RNA Maturation in Ehrlich Ascites Cells. *Cancer biochem. biophys.*, **1**, 57.

TOMASHEFSKY, P., HOMSY, Y. L., LATTIMER, J. U. & TANNENBAUM, M. (1976) A Murine Wilm's Tumor as a Model for Chemotherapy and Radiotherapy. *J. natn. Cancer Inst.*, **56**, 137.

WARREN, S. L. (1935) Preliminary Study of Effect of Artificial Fever upon Hopeless Tumor Cases. *Am. J. Roentgenol.*, **33**, 75.

WOLF, J. A. *et al.* (1968) Single *versus* Multiple Dose Actinomycin Therapy of Wilm's Tumor. *New Engl. J. Med.*, **279**, 290.

WOODSCOCK, D. M. & MANSBRIDGE, J. N. (1971) Rapidly Labelled Ribonucleoprotein Particles in Rat Liver Cytoplasm and their Relevance to the Transport of Messenger RNA. *Biochim. biophys. Acta*, **240**, 218.

YERUSHALMI, A. (1975) Cure of a Solid Tumor by Simultaneous Administration of Microwaves and X-ray Irradiation. *Radiat. Res.*, **64**, 602.

YERUSHALMI, A. (1976*a*) Treatment of a Solid Tumor by Local Simultaneous Hyperthermia and Ionizing Radiation: Dependence on Temperature and Dose. *Eur. J. Cancer*, **12**, 807.

YERUSHALMI, A. (1976*b*) Calculation of Intratumor Temperature in a Heated Superficial Tumor. *TIT J. Life Sci.*, **6**, 35.


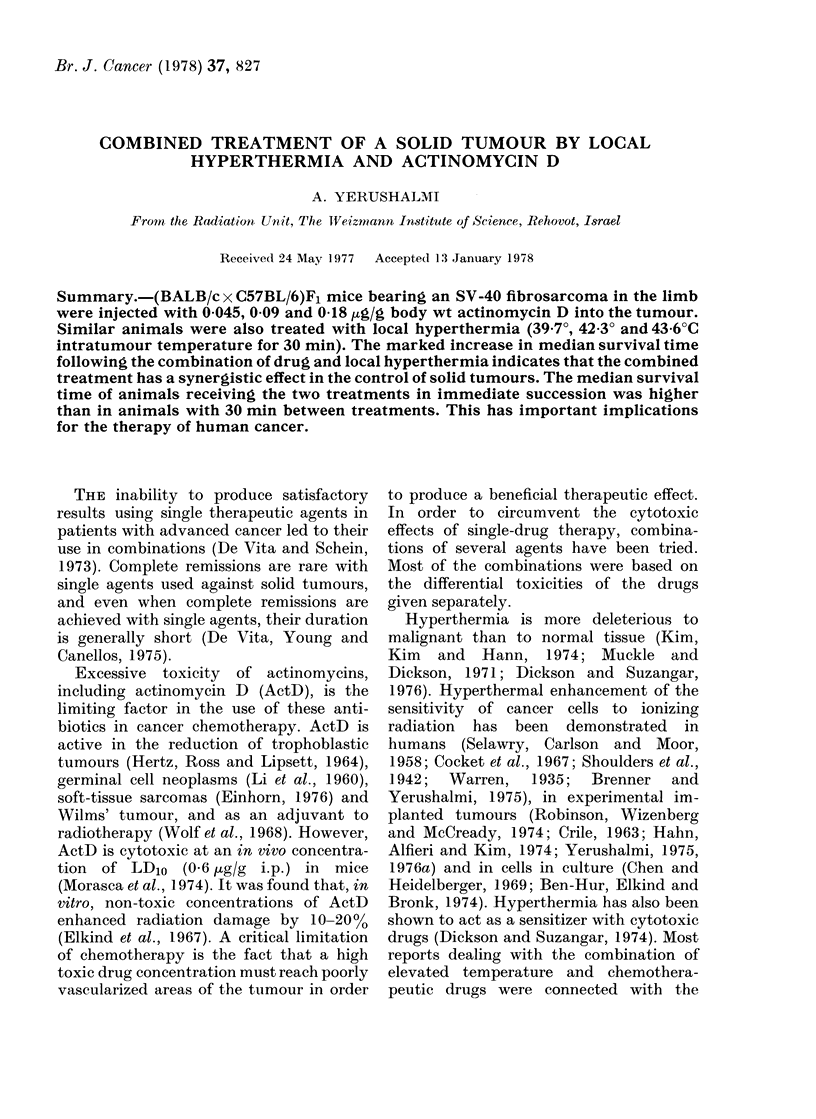

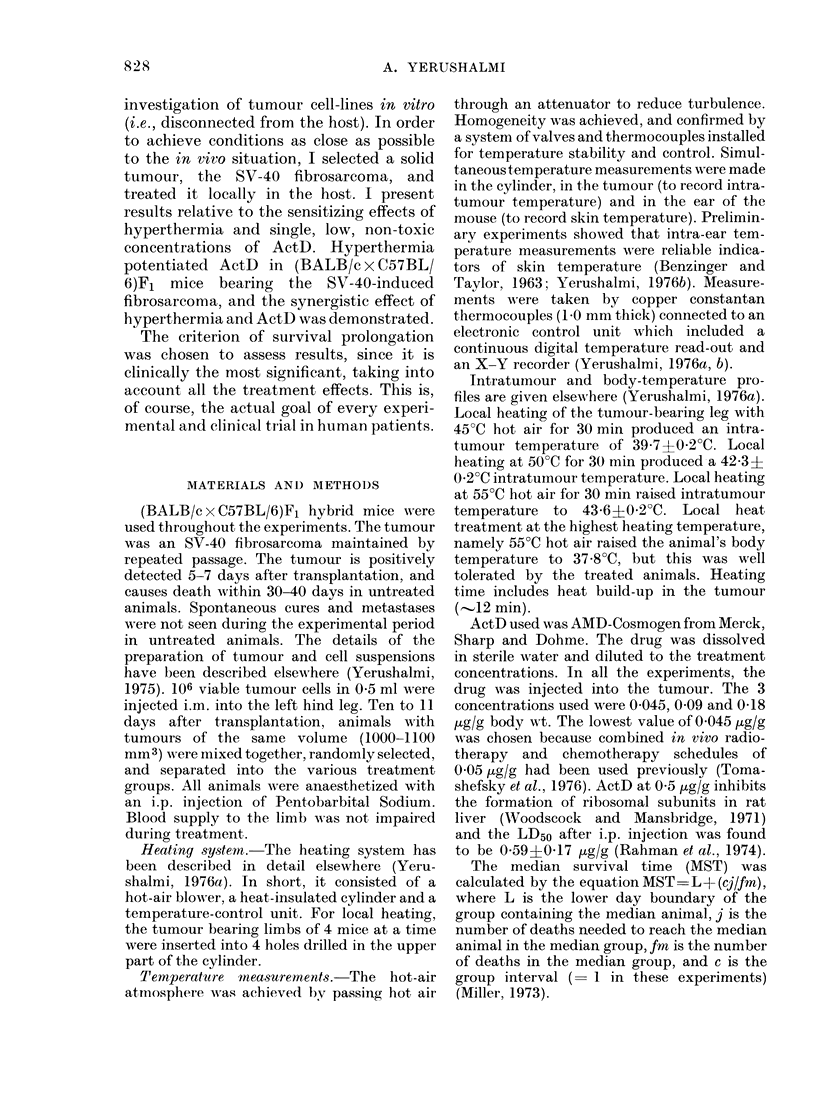

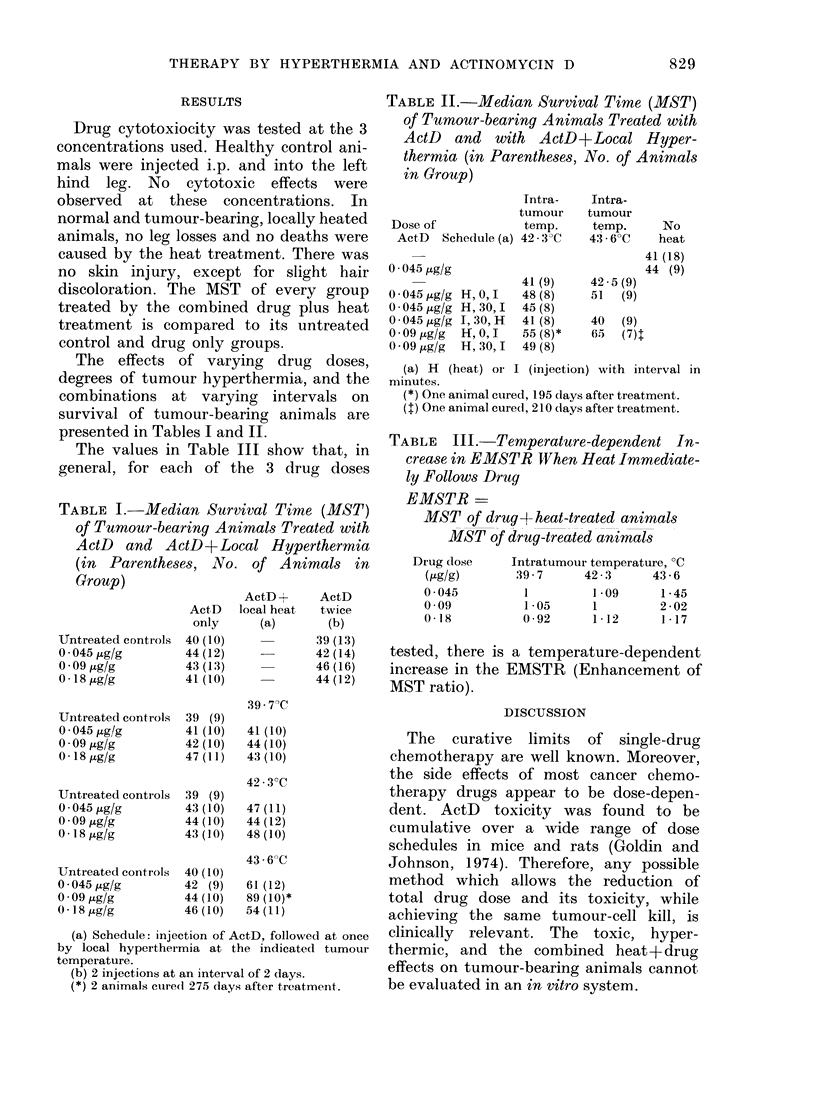

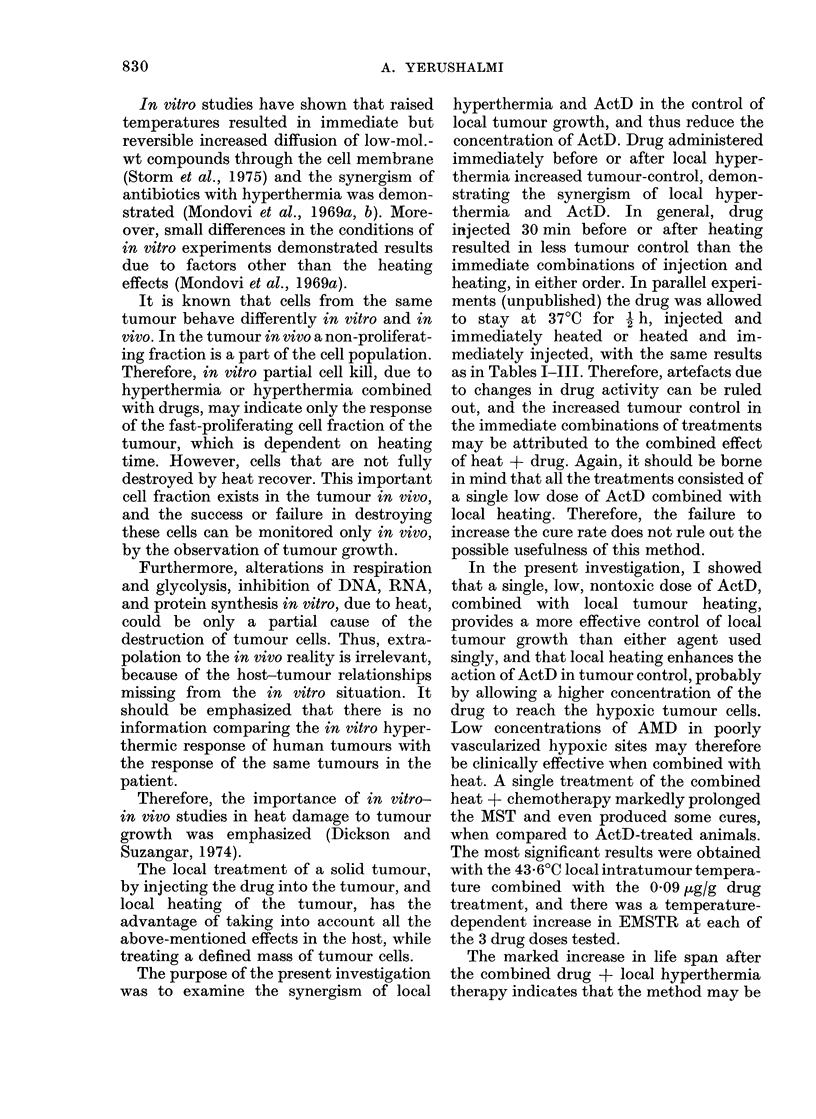

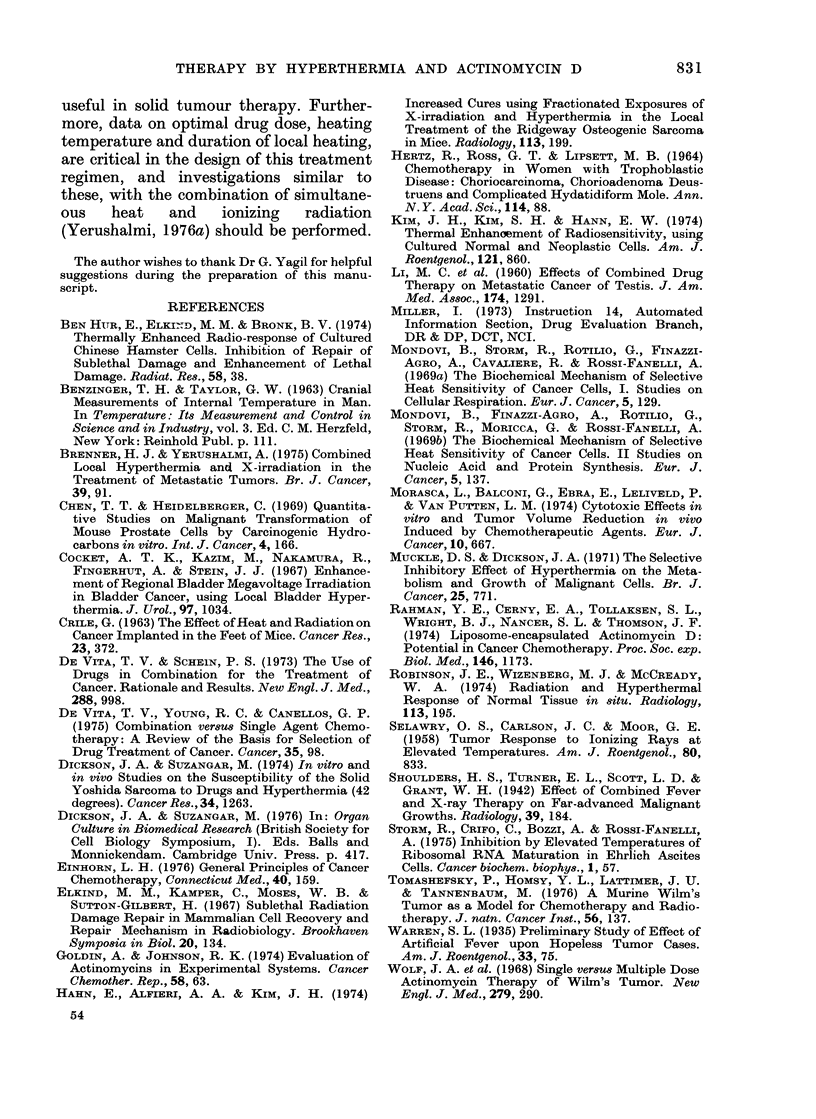

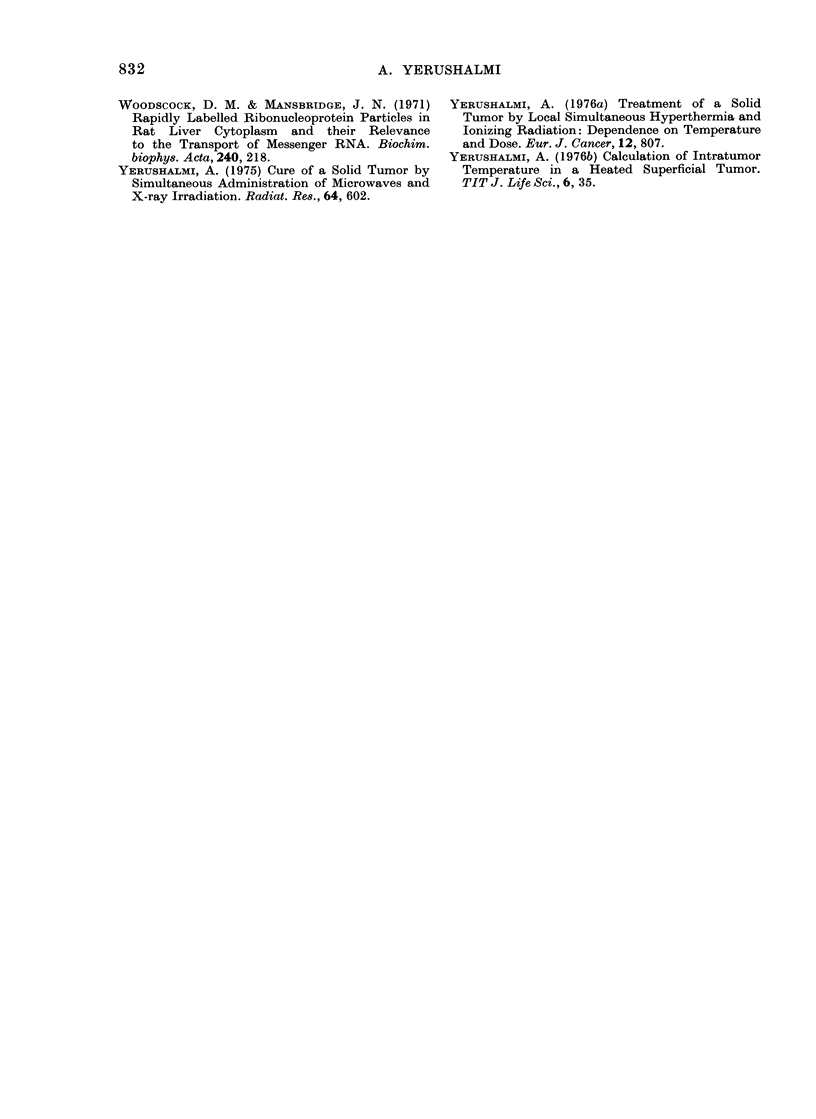


## References

[OCR_00597] CRILE G. (1963). The effects of heat and radiation on cancers implanted on the feet of mice.. Cancer Res.

[OCR_00584] Chen T. T., Heidelberger C. (1969). Quantitative studies on the malignant transformation of mouse prostate cells by carcinogenic hydrocarbons in vitro.. Int J Cancer.

[OCR_00590] Cockett A. T., Kazmin M., Nakamura R., Fingerhut A., Stein J. J. (1967). Enhancement of regional bladder megavoltage irradiation in bladder cancer using local bladder hyperthermia.. J Urol.

[OCR_00608] DeVita V. T., Young R. C., Canellos G. P. (1975). Combination versus single agent chemotherapy: a review of the basis for selection of drug treatment of cancer.. Cancer.

[OCR_00614] Dickson J. A., Suzangar M. (1974). In vitro-in vivo studies on the susceptibility of the solid Yoshida sarcoma to drugs and hyperthermia (42 degrees).. Cancer Res.

[OCR_00625] Einhorn L. H. (1976). General principles of cancer chemotherapy.. Conn Med.

[OCR_00636] Goldin A., Johnson R. K. (1974). Evaluation of actinomycins in experimental systems.. Cancer Chemother Rep.

[OCR_00641] Hahn E. W., Alfieri A. A., Kim J. H. (1974). Increased cures using fractionated exposures of X irradiation and hyperthermia in the local treatment of the Ridgway osteogenic sarcoma in mice.. Radiology.

[OCR_00655] Kim J. H., Kim S. H., Hahn E. (1974). Thermal enhancement of the radiosensitivity using cultured normal and neoplastic cells.. Am J Roentgenol Radium Ther Nucl Med.

[OCR_00661] LI M. C., WHITMORE W. F., GOLBEY R., GRABSTALD H. (1960). Effects of combined drug therapy on metastatic cancer of the testis.. JAMA.

[OCR_00678] Mondovì B., Finazzi Agrò A., Rotilio G., Strom R., Moricca G., Rossi Fanelli A. (1969). The biochemical mechanism of selective heat sensitivity of cancer cells. II. Studies on nucleic acids and protein synthesis.. Eur J Cancer.

[OCR_00671] Mondovì B., Strom R., Rotilio G., Finazzi Agrò A., Cavaliere R., Rossi Fanelli A. (1969). The biochemical mechanism of selective heat sensitivity of cancer cells. I. Studies on cellular respiration.. Eur J Cancer.

[OCR_00686] Morasca L., Balconi G., Erba E., Lelieveld P., Van Putten L. M. (1974). Cytotoxic effect in vitro and tumour volume reduction in vivo induced by chemotherapeutic agents.. Eur J Cancer.

[OCR_00693] Muckle D. S., Dickson J. A. (1971). The selective inhibitory effect of hyperthermia on the metabolism and growth of malignant cells.. Br J Cancer.

[OCR_00699] Rahman Y. E., Cerny E. A., Tollaksen S. L., Wright B. J., Nance S. L., Thomson J. F. (1974). Liposome-encapsulated actinomycin D: potential in cancer chemotherapy.. Proc Soc Exp Biol Med.

[OCR_00706] Robinson J. E., Wizenberg M. J., McCready W. A. (1974). Radiation and hyperthermal response of normal tissue in situ.. Radiology.

[OCR_00712] SELAWRY O. S., CARLSON J. C., MOORE G. E. (1958). Tumor response to ionizing rays at elevated temperatures: a review and discussion.. Am J Roentgenol Radium Ther Nucl Med.

[OCR_00730] Tomashefsky P., Homsy Y. L., Lattimer J. K., Tannenbaum M. (1976). A murine Wilms' tumor as a model for chemotherapy and radiotherapy.. J Natl Cancer Inst.

[OCR_00741] Wolff J. A., Newton W. A., Krivit W., D'Angio G. J. (1968). Single versus multiple dose dactinomycin therapy of Wilms's tumor. A controlled co-operative study conducted by the Children's Cancer Study Group A (formerly Acute Leukemia Co-operative Chemotherapy Group A).. N Engl J Med.

[OCR_00750] Woodcock D. M., Mansbridge J. N. (1971). Rapidly labelled ribonucleoprotein particles in rat liver cytoplasm and their relevance to the transport of messenger RNA.. Biochim Biophys Acta.

[OCR_00757] Yerushalmi A. (1975). Cure of a solid tumor by simultaneous administration of microwaves and x-ray irradiation.. Radiat Res.

